# An Operational Status Assessment Model for SF_6_ High-Voltage Circuit Breakers Based on IAR-BTR

**DOI:** 10.3390/s25133960

**Published:** 2025-06-25

**Authors:** Ningfang Wang, Yujia Wang, Yifei Zhang, Ci Tang, Chenhao Sun

**Affiliations:** 1 School of Electrical & Information Engineering, Changsha University of Science & Technology, Changsha 410114, China; m15717507668@163.com (N.W.); 202205040112@stu.csust.edu.cn (Y.Z.); 603598209@163.com (C.T.); 2 International College of Engineering, Changsha University of Science & Technology, Changsha 410114, China; wangyujia0724@outlook.com

**Keywords:** SF_6_ high-voltage circuit breakers, operational status assessment, spatiotemporally non-stationary factors, quantitative weighting method

## Abstract

With the rapid advancement of digitalization and intelligence in power systems, SF_6_ high-voltage circuit breakers, as the core switching devices in power grid protection systems, have become critical components in high-voltage networks of 110 kV and above due to their superior insulation performance and exceptional arc-quenching capability. Their operational status directly impacts the reliability of power system protection. Therefore, real-time condition monitoring and accurate assessment of SF_6_ circuit breakers along with science-based maintenance strategies derived from evaluation results hold significant engineering value for ensuring secure and stable grid operation and preventing major failures. In recent years, the frequency of extreme weather events has been increasing, necessitating a comprehensive consideration of both internal and external factors in the operational status prediction of SF_6_ high-voltage circuit breakers. To address this, we propose an operational status assessment model for SF_6_ high-voltage circuit breakers based on an Integrated Attribute-Weighted Risk Model Based on the Branch–Trunk Rule (IAR-BTR), which integrates internal and environmental influences. Firstly, to tackle the issues of incomplete data and feature imbalance caused by irrelevant attributes, this study employs missing value elimination (Drop method) on the fault record database. The selected dataset is then normalized according to the input feature matrix. Secondly, conventional risk factors are extracted using traditional association rule mining techniques. To improve the accuracy of these rules, the filtering thresholds and association metrics are refined based on seasonal distribution and the importance of time periods. This allows for the identification of spatiotemporally non-stationary factors that are strongly correlated with circuit breaker failures in low-probability seasonal conditions. Finally, a quantitative weighting method is developed for analyzing branch-trunk rules to accurately assess the impact of various factors on the overall stability of the circuit breaker. The DFP-Growth algorithm is applied to enhance the computational efficiency of the model. The case study results demonstrate that the proposed method achieves exceptional accuracy (95.78%) and precision (97.22%) and significantly improves the predictive performance of SF_6_ high-voltage circuit breaker operational condition assessments.

## 1. Introduction

As a pivotal component in modern power infrastructure, SF_6_ high-voltage circuit breakers have become the industry standard for high-current interruption in transmission systems operating at 110 kV and above [1]. These gas-insulated switching devices leverage SF_6_’s unique dielectric and arc-quenching properties to provide reliable circuit protection against both overload and short-circuit conditions [2]. Their widespread adoption stems from three fundamental advantages: (1) exceptional dielectric strength (approximately three times that of air at atmospheric pressure), (2) superior thermal conductivity for arc energy dissipation, and (3) chemical stability under normal operating conditions.

The operational architecture of SF_6_ HVCBs integrates four critical subsystems that collectively ensure interruption reliability: (i) the arc-extinguishing unit, featuring precision-engineered main/arcing contacts and nozzle geometries optimized for gas flow dynamics; (ii) a composite insulation system utilizing epoxy resin insulators and gas-insulated switchgear (GIS) enclosures; (iii) high-speed operating mechanisms (hydraulic/pneumatic/spring) capable of achieving contact separation within milliseconds; and (iv) real-time gas monitoring systems tracking density and moisture content as key performance indicators.

The interruption process exemplifies a sophisticated multi-physics phenomenon, progressing through three distinct yet interdependent stages. Initial contact separation generates an arc column reaching ~20,000 K, causing SF_6_ molecular dissociation into conductive S/F plasma. The puffer mechanism then drives supersonic gas flows (300–500 m/s) that simultaneously cool the arc channel through a convective heat transfer and turbulently disrupt plasma continuity. Crucially, SF_6_’s electronegativity facilitates rapid dielectric recovery via electron attachment reactions (SF_6_ + e^−^ → SF_6_^−^), achieving critical insulation strength within 1–2 μs post current-zero—a key factor preventing thermal resignation. This coordinated thermochemical-hydrodynamic process enables interruption ratings exceeding 63 kA while maintaining arcing durations below 15 ms, representing a ~40% performance improvement over conventional air-blast breakers [3].

However, in actual operation, the performance of SF_6_ high-voltage circuit breakers is influenced by both internal and external factors, leading to potential failure risks. On the one hand, internal factors such as gas leakage, reduced gas purity, aging, or wear of mechanical components over prolonged use degrade the performance of SF_6_ high-voltage circuit breakers [4,5]. On the other hand, external environmental changes interact with internal risk factors, exacerbating failures. Particularly under the increasing trend of extreme global climate conditions, this negative cycle becomes more pronounced, with extreme weather conditions even directly triggering failures. Evaluating and predicting the operational state of SF_6_ high-voltage circuit breakers, and taking maintenance actions in advance before failures occur, can mitigate their impact on power system operations, which is of great significance for the safe operation of those systems.

At present, many researchers have proposed numerous evaluation methods for the operational status of high-voltage circuit breakers, which can be categorized into five types based on technical principles and application characteristics. The first category is statistical model-based reliability analysis. For instance, Xiang Zhang et al. derived the failure probability, failure rate, and remaining service life of equipment and components by statistically investigating records of unfiled components and maintenance measures [6]. The second category relies on analyzing physical parameters to assess the operational status, where mechanical characteristics (e.g., closing and opening time, speed) [7] or electrical signals (e.g., arc, current characteristics) [8,9] are monitored to detect whether the circuit breaker operates normally. The third category is the fusion of signal analysis and intelligent algorithms, such as fault detection through vibration signal spectrum analysis [10], acoustic signal analysis [11], and contact monitoring using a breaker contact state recognition model based on vibration signals and improved neural networks [12]. Kuan Zhang et al. established a high-voltage circuit breaker fault diagnosis model based on LVQ neural networks and vibration signal analysis, improving accuracy through a combination of PCA-SSA-LVQ algorithms [13]. Xinyu Ye et al. optimized diagnostic accuracy and adaptability for small-sample data using a one-dimensional attention-based convolutional capsule neural network [14]. Yao et al. combined fractal technology and probabilistic neural networks (PNNs) to classify faults [15]. The fourth category involves data-driven and hybrid models that build empirical models using big data mining and machine learning. Yang et al. integrated fuzzy mathematics, expert systems, and machine learning techniques to establish predictive models [16]. Geng et al. optimized feature indicators and operational conditions to improve BP neural network performance [17]. Žarković et al. [18] employed artificial intelligence approaches incorporating cluster analysis (k-means, clustering tree) and artificial neural networks (ANNs) for health state assessment of SF_6_ circuit breakers. The fifth category of methods comprises knowledge-based approaches that rely on domain expertise and prior knowledge to construct models or decision systems. Diahovchenko et al. [19] applied fuzzy logic to evaluate the health status of SF_6_ circuit breakers and optimize maintenance priorities. Reference [20] integrated predictive data of dynamic contact resistance under varying current levels with domain expert knowledge to establish an expert system for assessing the contact erosion state, thereby providing explicit guidance for operation and maintenance decisions. Reference [21] utilized association rules to calculate fault risks of individual components within high-voltage circuit breaker subsystems, proposing both subsystem maintenance strategies considering fault correlations and multi-component combined maintenance strategies, which offer theoretical references for formulating maintenance strategies for high-voltage circuit breakers.

While the aforementioned methods have achieved certain progress in fault diagnosis of high-voltage circuit breakers as documented in the literature, they still exhibit significant limitations. Primarily, most existing studies demonstrate excessive reliance on single-dimensional data, lacking effective integration of multi-source heterogeneous data. Firstly, current research methodologies predominantly exhibit limitations in their one-dimensional data dependency and insufficient integration of multisource heterogeneous data. Conventional approaches, such as vibration signal analysis or traditional parameter monitoring, typically focus on singular parameter dimensions during modeling and prediction, thereby failing to account for the inherent complexity of actual power equipment. In contrast, Reference [22] proposes a more comprehensive fault prediction framework based on robust auto-associative kernel regression (AAKR), which systematically incorporates multiple critical parameters and still ignores the coupling effects of external environmental factors (e.g., temperature, humidity, mechanical vibration interference) on fault characteristics, leading to insufficient robustness of diagnostic models in complex working conditions. Although statistical models and data-driven methods utilize historical data, they do not explicitly model the dynamic relationship between environmental parameters and equipment aging, making them less adaptable to variable field conditions. Existing intelligent algorithms primarily optimize single-signal types without considering the synergistic analysis of mechanical and environmental parameters, leading to incomplete feature representation. Hybrid models and data-driven methods attempt to combine multiple techniques but fail to establish a cross-domain feature fusion mechanism, making it difficult to quantify the sensitivity of faults to environmental disturbances. Furthermore, association rule mining (ARM) approaches face inherent methodological limitations. First, the inherent variability in data quantity and the ambiguous definition of items frequently necessitate the lowering of mining threshold criteria to uncover quantitative association rules, resulting in the potential omission of significant patterns [23]. More critically, conventional ARM implementations typically employ static computational methodologies regardless of the application context, failing to account for spatiotemporal variations in operational conditions, and the critical influence of high-risk characteristic factors during low-probability seasonal periods on SF_6_ high-voltage circuit breaker performance.

To address these shortcomings in SF_6_ high-voltage circuit breaker operational status prediction, this study proposes a fault prediction method based on the Integrated Attribute-Weighted Risk Model Based on the Branch–Trunk Rule (IAR-BTR). This method comprehensively considers the impact of internal parameters and environmental factors on an SF_6_ high-voltage circuit breaker operation and can extract potential patterns of operational status from large-scale imbalanced data, thereby assessing whether failures will occur in the future. First, missing values in fault records are processed to filter reliable data. To facilitate feature management, different-dimensional feature matrices are standardized. Then, four types of association indicators are improved to encompass extreme factors and scenarios that are strongly correlated with SF_6_ high-voltage circuit breaker faults. In addition, BTR-based risk weight quantification is introduced to establish a relative risk weight calculation method that considers the interaction characteristics of various elements in SF_6_ high-voltage circuit breakers. This enables a more realistic and effective measurement of their varying impacts on overall system stability. The DFP-Growth algorithm is used for simulation. The effectiveness and flexibility of the proposed IAR-BTR model are verified through case studies, demonstrating its feasibility and adaptability for practical applications.

## 2. Establishment of the IAR-BTR Model

### 2.1. Establishment of Internal and Environmental Data Feature Repository

To enhance the accuracy of predicting the operational status of high-voltage circuit breakers, it is essential to acquire a sufficiently large and high-quality dataset, and to extract key metrics closely associated with operational performance. By mining the correlations between these metrics and the circuit breaker’s operational state, the underlying patterns for assessing the condition of SF_6_ high-voltage circuit breakers can be effectively identified. In the comprehensive assessment of SF_6_ high-voltage circuit breaker fault risks, both internal and external influencing factors must be considered in an integrated manner, as their combined effects jointly determine the overall risk level. Based on a systematic investigation and a thorough analysis of various influencing factors, an internal–environmental coupling strategy was adopted for input feature selection; the internal and external factors selected for input feature construction are listed in Table 1.

#### 2.1.1. Missing Data Processing

In practical datasets, missing data features are often unavoidable, arising from incorrect recording methods, equipment failures, or other issues encountered during the data acquisition process. In the dataset used for circuit breaker condition prediction, the presence of missing values is likely to adversely affect the model’s accuracy and reliability. Therefore, handling missing values in advance is a critical step in data preprocessing.

A common approach to addressing missing data is dropping missing values [24], where samples or features containing missing entries are directly removed. This method offers the advantage of simplicity while maintaining the internal consistency of the dataset; however, it also carries the risk of information loss. To maximize the utilization of the existing database and minimize the impact of missing values on model performance, this study adopts a drop strategy for features with a missing data proportion greater than 5%.

#### 2.1.2. Standardization of Internal–Environmental Heterogeneous Data Matrix

To facilitate the management of features and the identification of attributes associated with the operational status of circuit breakers, it is necessary to standardize the management of features with diverse properties. Without such standardization, the subsequent data analysis workload would significantly increase and could even lead to the loss of feature data. Additionally, the heterogeneity among different features may cause imbalances in the influence of certain continuous features on prediction outcomes during model training.

To address this issue, standardized processing of internal–environmental heterogeneous data is typically employed to harmonize feature types and visualize complex data structures, thereby improving the stability and accuracy of the model. In this study, externally collected data were consolidated into the data processing space, and continuous features within the dataset were discretized to ensure uniformity and enhance model performance. Let $L=\left\{c_{1},c_{2},\ldots ,c_{i},\ldots c_{m}\right\}$ denote the set of labels corresponding to the m fault records. Let $A=\left\{a_{1},\ldots ,a_{j},a_{j+1},\ldots a_{n}\right\}$ represent the set containing all internal and environmental input features of SF_6_ high-voltage circuit breakers, $\mathrm{where}\;a_{j}$ is an individual input feature variable. Each input feature $a_{j}$ is composed of a group of factors $\left\{b_{j,1},\ldots ,b_{j,k},\ldots b_{j,l}\right\}$, which collectively characterize whether the operational state of the SF_6_ high-voltage circuit breaker is normal. Let $P=\left\{p_{1},p_{2},\ldots ,p_{i},\ldots p_{m}\right\}$ denote the set of m target variables, where $p_{i}$ represents a specific target variable. Let $S=\left\{s_{1},s_{2},\ldots ,s_{i},\ldots s_{m}\right\}$ denote the set containing seasonal information corresponding to each fault record, where each element in S is one of the four seasons: spring, summer, autumn, or winter. The constructed matrix consists of four parts: the label vector, the input feature matrix, the target vector, and the time (season) vector. The first row of the matrix corresponds to the data header. Based on the above definitions, the matrix structure is formulated as follows:

(1) $$L={\left\{c_{1},c_{2},\ldots ,c_{i},\ldots c_{m}\right\}}^{T}$$

(2) $$A=\left[\begin{array}{cccccc} a_{1} & \ldots & a_{j} & a_{j+1} & \ldots & a_{n}\\ b_{11} & \ldots & b_{1j} & b_{1j+1} & \ldots & b_{1n}\\ \vdots & \ddots & \vdots & \vdots & \ddots & \vdots \\ b_{i1} & \ldots & b_{ij} & b_{ij+1} & \ldots & b_{in}\\ \vdots & \ddots & \vdots & \vdots & \ddots & \vdots \\ b_{m1} & \ldots & b_{mj} & b_{mj+1} & \ldots & b_{mn} \end{array}\right]$$

(3) $$P={\left\{p_{1},p_{2},\ldots ,p_{i},\ldots ,p_{m}\right\}}^{T}$$

(4) $$S={\left\{S_{1},S_{2},\ldots ,S_{i},\ldots ,S_{m}\right\}}^{T}$$

In this formulation, each element $c_{1},c_{2},\ldots ,c_{i},\ldots c_{m}$ in the label vector L represents the fault label corresponding to each fault record. In the input feature matrix A, the first row $a_{1},\ldots ,a_{j},a_{j+1},\ldots a_{n}$ denotes the names of the input features associated with the operational status of SF_6_ high-voltage circuit breakers. Specifically, the columns from $a_{1}$ to $a_{j}$ correspond to external environmental features, while the columns from $a_{j+1}$ to $a_{n}$ correspond to internal features of the circuit breaker. For each fault record, the feature variables are recorded starting from the second row. Here, $b_{ij}$ denotes the feature factor corresponding to the fault record with label $c_{i}$ and feature $a_{j}$. Multiple features jointly determine the corresponding target variable $p_{i}$ in the target vector P. The element $S_{i}$ in the set S represents the season associated with the fault labeled $c_{i}$, where $S_{i}\in \left\{S_{Sp},S_{Su},S_{Au},S_{Wi}\right\}$ correspond to spring, summer, autumn, and winter, respectively.

The data processing matrix $DF_{O}$ for circuit breaker operational status prediction, constructed with n attributes, can be expressed as:

(5) $$DF_{O}=\left[L\;A\;P\;S\right]$$

### 2.2. Two-Stage Extraction of Risk Factors Based on Attribute Importance

#### 2.2.1. Identification of Conventional Risk Factors

The occurrence of one event triggering the occurrence of another indicates a certain degree of association between the two events, which are referred to as associated events. Association rules are commonly used to discover the corresponding relationships between events [25]. In this study, association rule mining is applied to explore the relationships between the internal and environmental conditions of SF_6_ high-voltage circuit breakers and their fault occurrences. Item sets are used to represent the associations between events. For the fault records of SF_6_ high-voltage circuit breakers, let $E=\left\{e_{1},e_{2},e_{3}\ldots \right\}$ denote the set containing all input environmental feature factors. Let O represent the antecedent feature set, which is a subset of E, and P denote the consequent target variable. Then, an association rule can be formulated as: O→P.

Among the various associations that exist between events, only those satisfying specific constraint conditions are considered meaningful for research purposes. Therefore, to effectively define association rules, it is necessary to first introduce association filtering diagnostic metrics. If a rule satisfies all the predefined threshold values of filtering metrics, it can be inferred that when factor O occurs, the outcome P is highly likely to occur as well. In this study, support, confidence [26], lift, and the Jaccard coefficient are selected as filtering metrics for evaluating association rules.

Support is used to evaluate the probability of the occurrence of an item set among all possible events. It represents the proportion of the number of antecedent feature factor item sets relative to the total number of consequent target variables in the input database $DF_{O}$. The calculation formula is given as follows:

(6) $$spt\left(O\right)=\frac{\sum Ⅱ\left[c\in DF_{O}]\cdot Ⅱ[O\subseteq c\right]}{Nb\left(DF_{O}\right)}=P\left(O\cup P\right)$$

In the above formulation, $spt\left(O\right)$ denotes the support of event O. The indicator funtion Ⅱ serves as a binary operator: $Ⅱ\left[c\in DF_{O}\right]\cdot Ⅱ\left[O\subseteq c\right]=1$ if and only if the database $DF_{O}$ contains event O and the antecedent feature event has a corresponding label in the input database; otherwise, the result is 0. $Nb\left(DF_{O}\right)$ represents the total number of event transactions contained in the input database $DF_{O}$. According to the rule filtering criteria, the support of a valid association rule must be no less than a specified minimum support threshold. A higher support value indicates that the corresponding rule occurs more frequently in the fault records of SF_6_ high-voltage circuit breakers.

Confidence is used to measure the reliability of an association rule. Specifically, for an association rule of the form O→P, confidence is defined as the ratio of the number of event feature item sets in $DF_{O}$ that contain both O and P to the number of event feature item sets that contain O alone. The calculation formula is as follows:

(7) $$cf\left(O\to P\right)=\frac{P\left(O,P\right)}{P\left(O\right)}=\frac{spt\left(O\cap P\right)}{spt\left(O\right)}$$

Similarly to the support metric, the confidence of an association rule must not be lower than the predefined minimum confidence threshold to be considered reliable. A higher confidence value indicates a stronger association between the rule and the occurrence of faults in SF_6_ high-voltage circuit breakers.

The lift reflects the degree of association between antecedent and consequent events and serves as a measure of rule validity. By evaluating whether the observed association exceeds what would be expected by random chance, the lift effectively eliminates spurious strong associations and filters out invalid rules. The calculation formula for lift is given as follows:

(8) $$Lf\left(O\to P\right)=\frac{P\left(P|O\right)}{P\left(P\right)}=\frac{spt\left(O\to P\right)}{spt\left(O\right)spt\left(P\right)}$$

The calculation results of the lift are used to screen practically useful association rules by employing a threshold of 1. Specifically, only association rules with Lf>1 are considered effective and strongly associated; if Lf≤1, it indicates that item sets O and P are either uncorrelated or mutually exclusive, and thus such rules are excluded from consideration.

The Jaccard coefficient is employed to measure the similarity between item sets and to analyze the strength of associations in sparse datasets. It is defined as the ratio of the number of events where both items occur together to the number of events containing at least one of the item sets. The calculation formula is expressed as follows:

(9) $$J\left(O\to P\right)=\frac{Nb\left(O\cap P\right)}{Nb\left(O\cup P\right)}$$

The Jaccard coefficient ranges from 0 to 1, with a higher value indicating greater similarity. A coefficient of 0 signifies complete mutual exclusivity, while a coefficient of 1 indicates complete overlap.

#### 2.2.2. Identification of Spatiotemporal Non-Stationary Risk Factors

Investigation revealed that a certain class of feature factors exhibit significant spatiotemporal variability in their probability of triggering SF_6_ high-voltage circuit breaker faults. These factors may have an extremely low occurrence probability during certain periods, making it difficult to identify using traditional Association Rule Mining (ARM) algorithms. However, once they occur, they can pose a substantial threat to the operational stability of SF_6_ high-voltage circuit breakers. Such low probability but high-risk feature factors, which demonstrate seasonal and spatial non-stationarity, are referred to as Spatiotemporally Non-Stationary Risk Factors (SNSRFs). In regions with pronounced seasonal environmental variations, faults induced by SNSRFs tend to display uneven seasonal distributions. For instance, in areas characterized by monsoon climates, lightning-induced faults are more prevalent in the summer due to frequent thunderstorms, whereas such faults are rare in winter. Given the unique characteristics of SNSRF-induced faults, it is necessary to perform a dedicated analysis of SNSRFs to enhance the accuracy and robustness of the fault risk assessment system.

To effectively mine SNSRFs, rare association item sets are first separated from frequent item sets for independent analysis. A typical association rule involving both common and rare variables can be represented in the following form:

(10) $$O_{g}+O_{r}\to P$$

where $O_{g}$ represents the set of high-frequency association item sets, while $O_{r}$ denotes the set of rare association item sets.

Since not all factors exhibiting temporal imbalance characteristics qualify as SNSRFs, it is necessary to further mine SNSRFs from the rare factors identified. Applying traditional association rule mining (ARM) models to the prediction of SF_6_ high-voltage circuit breaker faults, conventional ARM algorithms use the same fixed importance score calculation methods and filtering thresholds for all fault factors, regardless of their temporal distribution. This approach tends to assign low importance scores to fault factors occurring during rare periods, potentially causing them to fall below the predefined filtering diagnostic threshold established for an annual cycle. Consequently, fault factors associated with rare periods may be prematurely filtered out. This limitation leads to the inadvertent exclusion of SNSRFs associated with low-probability periods, resulting in the underutilization of valuable data resources. Therefore, it is essential to refine the association indicator thresholds and importance score calculation methods by incorporating temporal attribute information, enabling a more comprehensive evaluation of both the fault factors and the periods in which they occur.

The threshold adjustment for association indicators primarily considers the variability in the fault-triggering frequency of the SNSRF across different periods. To ensure that the influence of the SNSRF during rare periods is not overlooked, the year is first divided into four typical periods—spring, summer, autumn, and winter—based on seasonal differences in environmental conditions. An indicator function is then employed to calculate the number of SF_6_ high-voltage circuit breaker faults occurring in each season within the database, and the number of faults in the season with the highest fault frequency is identified. The calculation method is as follows:

(11) $$I_{D}=Ⅱ\left[c_{i}\in DF_{O}\left(i,1\right)\right]$$

(12) $$I_{S}=Ⅱ\left[DF_{O}\left(i,n+3\right)=S_{\left(s\right)}\right]$$

(13) $$C_{1}=\overset{m+1}{\underset{i=2}{\sum }}I_{D}\cdot I_{S}$$

In this context, i denotes the row index of an SF_6_ high-voltage circuit breaker fault record within the input database $DF_{O}$. The term $S_{\left(s\right)}$ represents one of the four seasons: spring, summer, autumn, or winter. The indicator function $I_{D}$ determines whether a fault record belongs to the database $DF_{O}$; it returns 1 if the record is included in $DF_{O}$, and 0 otherwise. Similarly, the indicator function $I_{S}$ determines whether a fault record occurs in a specified season. $C_{1}$ denotes the number of SF_6_ high-voltage circuit breaker fault records in the database for each season, where $C_{1}\in \{C_{1_{Sp}},C_{1_{Su}},C_{1_{Au}},C_{1_{Wi}}\}$ correspond to spring, summer, autumn, and winter, respectively.

Based on the four filtering indicators—support, confidence, lift, and the Jaccard coefficient—association status filtering thresholds are set for each typical seasonal period according to the distribution of faults across seasons. An improved minimum support threshold is adopted as a filtering criterion to extract useful association rules, retaining only those item sets whose support is greater than or equal to the minimum support value. Similarly, a minimum confidence threshold is established to assess the reliability of the association rules; rules with confidence below the threshold are filtered out. The expressions for setting the corresponding filtering thresholds are given as follows:

(14) $$C_{2}=\max\{C_{1_{Sp}},C_{1_{Su}},C_{1_{Au}},C_{1_{Wi}}\}$$

(15) minsptO=minspt0·C1C2

(16) $$mincf\left(O\right)=mincf^{0}$$

(17) $$minlf\left(O\right)=minlf^{0}\cdot \frac{\sum_{i=2}^{m+1}I_{D}\cdot I_{s}\cdot Ⅱ\left[DF_{O}\left(i,n+2\right)=P_{t}\right]}{\sum_{i=2}^{m+1}I_{D}\cdot I_{s.max}\cdot Ⅱ\left[DF_{O}\left(i,n+2\right)=P_{t}\right]}$$

(18) $$minJ\left(O\right)=minJ^{0}\cdot \frac{\sum_{i=2}^{m+1}I_{D}\cdot I_{s}\cdot Ⅱ\left[DF_{O}\left(i,n+2\right)=P_{t}\right]}{\sum_{i=2}^{m+1}I_{D}\cdot I_{s.max}\cdot Ⅱ\left[DF_{O}\left(i,n+2\right)=P_{t}\right]}$$

$C_{2}$ represents the number of fault occurrences during the season with the highest SF_6_ high-voltage circuit breaker fault frequency in the database for a given year. $I_{s.max}$ is used to determine whether a fault record occurred during the season with the maximum fault frequency. $P_{t}$ denotes a specific outcome within the fault handling results. $minspt^{0},mincf^{0},minlf^{0},minJ^{0}$ represent the initially set threshold values for association rule states. Fault events are filtered based on the importance of attributes in each timely period; attributes falling below the corresponding threshold are eliminated, and only conventional risk feature factors that meet or exceed the threshold are retained.

In designing the calculation rules for the importance of each attribute across different time periods, this study improves upon traditional methods by introducing the concept of conditional probability. When predicting the operational status of SF_6_ high-voltage circuit breakers, the distribution of rare factors under different environmental characteristics is analyzed to calculate the scores of various types of rare factors. Based on the distribution of faults involving rare factors, the SNSRFs strongly associated with the target are further mined from the rare data set. Let f denote a function representing the inclusion relationship between two item sets. Following improvements in the standard scoring method for association indicators, the revised indicator scoring formula is given as:

(19) $$R=Ⅱ\left[DF_{O}\left(i,j\right)\in O_{r}\neq \emptyset \right]$$

(20) $$S_{j\left(spt\right)}=\frac{\sum_{i=2}^{m+1}I_{D}\cdot Ⅱ\left[f\left(O_{g},DF_{O}\left(i,M_{d}\right)\right)\neq \emptyset \right]\cdot R}{\sum_{i=2}^{m+1}I_{D}\cdot R}$$

(21) $$S_{j\left(cf\right)}=\frac{\sum_{i=2}^{m+1}I_{D}\cdot R\cdot Ⅱ\left[f\left(O_{g},DF_{s}\left(i,M_{d}\right)\right)\neq \emptyset ]\cdot Ⅱ[DF_{O}\left(i,n+2\right)=P_{t}\right]}{\sum_{i=2}^{m+1}I_{D}\cdot R}$$

(22) $$S_{j\left(lf\right)}=\frac{\sum_{i=2}^{m+1}I_{D}\cdot Ⅱ\left[f\left(O_{g},DF_{O}\left(i,M_{d}\right)\right)\neq \emptyset \right]\cdot R}{\sum_{i=2}^{m+1}I_{D}\cdot R\cdot Ⅱ\left[DF_{O}\left(i,n+2\right)=P_{t}\right]}\cdot S_{j\left(cf\right)}$$

(23) $$S_{j\left(J\right)}=\frac{\sum_{i=2}^{m+1}I_{D}\cdot Ⅱ\left[f\left(O_{g},DF_{O}\left(i,M_{d}\right)\right)\neq \emptyset \right]\cdot R}{\sum_{i=2}^{m+1}I_{D}\cdot R\cdot Ⅱ\left[f\left(O_{g},DF_{O}\left(i,M_{d}\right)\right)\neq \emptyset ]\cdot Ⅱ[DF_{O}\left(i,n+2\right)=P_{t}\right]}$$

where R determines whether the item belongs to a rare item set, and $f\left(O_{g},DF_{O}\left(i,M_{d}\right)\right)$ indicates whether the item set $O_{g}$ is contained within the item set $DF_{O}\left(i,M_{d}\right)$.

#### 2.2.3. Algorithm Implementation Process

In this study, the DFP-Growth algorithm is employed, which applies to a drop missing values strategy during the preprocessing stage. Specifically, data records with a feature missing rate greater than 5% are removed to ensure the completeness of the sample feature vector set. Based on the filtered dataset, compared to the traditional FP-Growth algorithm [27], the DFP-Growth algorithm adopts a heuristic strategy for selecting conditional pattern bases. This approach eliminates the need to scan all frequent item sets individually, thereby reducing the number of recursive calls and requiring the construction of only a single FP-tree. Table 2 presents the comparative performance metrics of the DFP-Growth algorithm versus the FP-Growth algorithm in terms of runtime and memory consumption. The DFP-Growth algorithm improves computational efficiency and reduces memory consumption, demonstrating better scalability, particularly for mining tasks on large-scale datasets or in resource-constrained environments.

### 2.3. Integrated Attribute-Weighted Risk Model Based on Branch–Trunk Rule (IAR-BTR)

The impact of the fault factors identified earlier on the operational status of SF_6_ high-voltage circuit breakers needs to be quantitatively assessed. Directly calculating the contribution of each fault factor can reveal the overall trend and magnitude of risk variation. However, in practical scenarios, fault factors are often interdependent rather than completely independent, making it difficult to determine the absolute influence of each factor on the overall system. To address this issue, this study considers the interactions and intrinsic correlations among fault factors and derives the overall system risk by calculating the dependency-based weighted contribution of each factor. A Birnbaum importance measure [28], which integrates the relative strength of component positions within the system reliability structure, is employed to evaluate the weight of each factor. Based on a branch–trunk structural importance metric, a Branch–Trunk Rule is designed to quantify the relative contributions of different factors.

The operational impact $X_{A}$ of a critical feature on the system is composed of two mutually exclusive outcomes: $X_{A}=1$ indicates system failure, while $X_{A}=0$ represents normal system operation. Under this definition, the state of the critical factor satisfies the following probabilistic condition:

(24) $$P\left(X_{A}=0\right)+P\left(X_{A}=1\right)=1$$

For each risk feature, a set of parts describing the status of the associated risk factors is constructed. The actual status of each part is represented as a discrete multi-valued variable, denoted by $x_{j,k}\in \{0,1,\cdots ,L_{j,k}\}$, where 0 indicates a completely normal state, and $L_{j,k}$ represents the highest failure level for the (j,k)-th part. The degree of failure increases with the numerical value of the part state.

To facilitate data processing and analysis, the part states are normalized by classifying them into two categories: normal and failure. The normalized failure function is defined as follows:

(25) $$\Phi_{j,k}\left(x_{j,k}\right)=\frac{x_{j,k}}{L_{j,k}}$$

In this context, $x_{j,k}=0$ indicates a fully normal state, while $x_{j,k}=L_{j,k}$ denotes complete failure. States with values between 0 and 1 represent degraded conditions. A part judgment threshold η=0.5 is introduced, such that when $\Phi_{j,k}\left(x_{j,k}\right)<0.5$, the part is considered to be in a normal state, whereas when $\Phi_{j,k}\left(x_{j,k}\right)\geq 0.5$, the part is deemed to be in a failed state.

All parts associated with the system’s multi-layer risk features are organized into a matrix. The structural tree importance measure conducts pairwise logical analysis of the states of contributing factors within the system, examining the potential outcomes induced by different fault states. Based on this analysis, the overall system security is evaluated. The corresponding mathematical expression is given as follows:

(26) $$Q_{jk}=2^{-n}\overset{n}{\underset{k=1}{\sum }}\overset{l}{\underset{j=1}{\sum }}\left(X_{jk}-\bar{X_{jk}}\right)$$

Here, $X_{jk}$ and $\bar{X_{jk}}$ represent two possible states of the part expression system for the k-th part within the j-th feature, respectively. As the value of $Q_{jk}$ increases, the overall system risk correspondingly rises.

**Figure 1:**
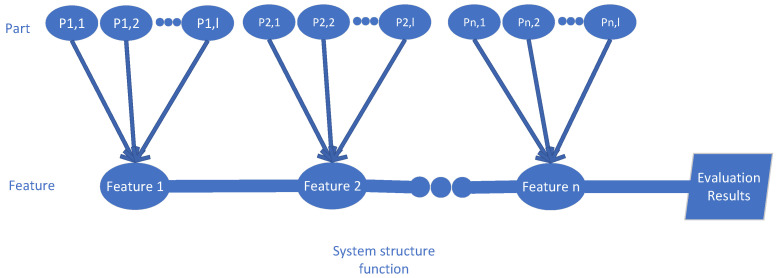
Reliability tree diagram.

Subsequently, the structure function for the proposed impact weight evaluation model is constructed. In Figure 1, a Reliability Tree Diagram (RTD) is employed to describe the relative positions and logical relationships among all features, as well as the parts they contain. Based on the principle of cut sets, the branch–trunk set is defined as a collection of parts whose simultaneous failure leads to system failure. A minimal branch–trunk set refers to the smallest such set: if any part is removed, the remaining parts no longer constitute a branch–trunk set. In this study, the minimal branch–trunk set B represents the minimal collection of parts within each feature that ensures the system’s reliability would be impacted. If $B_{1},B_{2}\ldots \ldots ,B_{\alpha },\ldots ,\;B_{g}$ denote the minimal branch–trunk sets for a given system, the structure function can be expressed as follows:

(27) $$X=-\overset{g}{\underset{\alpha =1}{\prod }}[1-\underset{\left(j,k\right)\in B_{\alpha }}{\prod }\Phi_{j,k}\left(x_{j,k}\right)]+1$$

where $\left[g\right]$ denotes the set of all minimal branch sets within the system, and $B_{\alpha }$ represents a specific minimal branch set. When all parts within a branch set reach their maximum failure states, the structure function $\Phi_{j,k}=1$, the internal product equals 1, and the system is ultimately determined to have failed. Conversely, when all components are in normal condition, $\Phi_{j,k}=0$, the internal product equals 0, and the system is judged to be operating normally. If some parts are in a degraded state, the output of the structure function lies between 0 and 1, indicating that the system is operating in a critical state.

As illustrated in the RTD, a feature vector consists of multiple branch part vectors connected in parallel, while multiple feature vectors are connected in series to ultimately form the trunk structure of the system, thereby producing a comprehensive evaluation result. In the reliability tree model, a fault in the SF_6_ high-voltage circuit breaker implies that at least one unsafe branch exists among the features constituting the trunk; that is, within the relevant features of a branch set, there must be a part state that leads to system failure. To reflect the role of each part in the structure function and to quantify its contribution to the overall system failure, a part failure weight calculation formula is defined as follows:

(28) $$w_{j,k}=\overset{m}{\underset{i=2}{\sum }}\frac{\sum_{i=2}^{m+1}I_{D}\cdot Ⅱ\left[DF_{s}\left(i,j\right)=b_{j,k}\right]}{\sum_{i=2}^{m+1}I_{D}\cdot Ⅱ\left[DF_{s}\left(i,j\right)\in a_{j}\right)]}$$

The overall fault weight calculation formula constructed from the feature vectors can be expressed as:

(29) $$X=\overset{n}{\underset{j=1}{\prod }}B_{\alpha }$$

where $B_{\alpha }$ represents the attribute contribution of each feature to the fault assessment.

The final function for evaluating fault risk can thus be expressed as:

(30) $$X=\overset{n}{\underset{j=1}{\prod }}[1-\overset{l}{\underset{k=1}{\prod }}(1-\overset{m}{\underset{i=2}{\sum }}\frac{\sum_{i=2}^{m+1}I_{D}\cdot Ⅱ\left[DF_{O}\left(i,j\right)=b_{j,k}\right]}{\sum_{i=2}^{m+1}I_{D}\cdot Ⅱ\left[DF_{O}\left(i,j\right)\in a_{j}\right]})]$$

## 3. The Operation Procedure of the IAR-BTR Model

By applying the above methods, this study establishes the Integrated Attribute-Weighted Risk Model Based on the Branch–Trunk Rule (IAR-BTR) to evaluate the operational status of SF_6_ high-voltage circuit breakers. The specific implementation process is as follows:

1. Fault records with missing feature proportions exceeding 5% are removed to obtain the sample dataset. The remaining records are subjected to matrix standardization based on the internal and environmental input features of SF_6_ high-voltage circuit breakers, and a feature repository is established;
2. For each input feature $a_{j}$ in the training dataset, conventional risk factors are identified by applying association-filtered diagnostic score calculations (Equations (6)–(9)) across all elements contained in $a_{j}$;
3. Using the improved association thresholding method (Equations (15)–(18)), the association rule sets are divided into a frequent item set $O_{g}$ and a rare item set $O_{r}$. Based on the improved association score calculation (Equations (18)–(23)), spatiotemporal non-stationary risk factors are mined from the rare item set $O_{r}$. to characterize their impacts on the operational status of SF_6_ high-voltage circuit breakers;
4. Steps 1–3 are repeated sequentially for each environmental feature in the training dataset;
5. Normalize each part’s state and the fault weight $w_{\left\{j,k\right\}}$ for each part computed using Equation (28), and subsequently, the fault risk value X of the SF_6_ high-voltage circuit breaker is derived using Equation (30);
6. Based on Steps 1–5, the fault risk value for each fault record in the dataset is calculated and normalized within the range [0, 1], where 0 indicates an impossible occurrence and 1 indicates a certain occurrence of failure;
7. The predicted fault risk weights are compared with the actual fault records (labeled as 0 or 1) in the test dataset to validate the performance of the proposed IAR-BTR prediction model.

**Figure 2:**
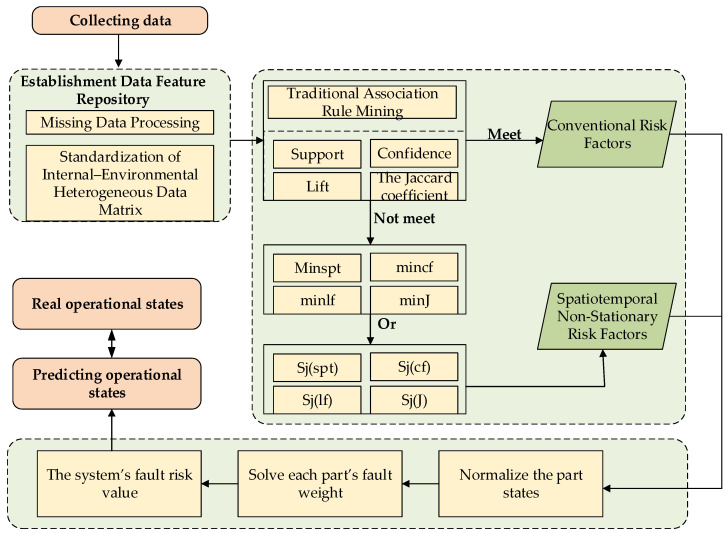
Flowchart.

Based on the above steps, the implementation flowchart of the IAR-BTR model is shown below in Figure 2:

## 4. Empirical Case Study

### 4.1. Test Data

In this study, experimental validation was conducted using SF_6_ high-voltage circuit breaker records collected from power plants in a province of China. After missing value processing, a total of 521 sample records were obtained. The dataset was divided into training and testing subsets with a ratio of 7:3, where 70% of the records were used for training and 30% were reserved for testing.

### 4.2. Experimental Environment

The experimental environment consisted of two computing platforms: a high-performance with an Intel Core i7-7700 quad-core processor (3.6 GHz, 8 threads), 32 GB RAM, and 2 TB storage running 64-bit Windows, alongside a portable laptop configuration featuring an Intel Core i5-2450 M dual-core processor (2.5 GHz, 4 threads), 8 GB RAM, and 500 GB storage under the same 64-bit Windows OS. All computational procedures were executed in MATLAB R2022a (Math Works) as the unified software environment, ensuring consistent experimental conditions across both hardware platforms while maintaining the necessary computational capacity for algorithm benchmarking.

### 4.3. Validation Method

To validate the superiority of the proposed IAR-BTR method, this study selected several widely used classification models, including LGBM [29], Tabular Neural Network [30,31], and Naive Bayes (NB) [32], to establish fault prediction models for SF_6_ high-voltage circuit breakers and compare their performance with that of the IAR-BTR method. A 10-fold cross-validation strategy was employed to ensure the reliability and robustness of the evaluation results. Through repeated testing, the optimal values of the key hyper parameters for each model are presented in Table 3.

The performance of the models was comprehensively assessed using four metrics: Receiver Operating Characteristic (ROC) curve [33], Precision-Recall (PR) curve [34], Kolmogorov–Smirnov (KS) curve [35], Detection Error Tradeoff (DET) curve [36], Confusion Matrix [37], Accuracy and Precision.

The evaluation metrics are described as follows:

1. ROC Curve and AUC Metric:

The ROC curve reflects the overall classification performance of a model across different decision thresholds. Based on the ROC curve, the Area Under the Curve (AUC) is calculated as a quantitative metric; a higher AUC value indicates better overall classification accuracy.

2. PR Curve:

The PR curve measures the relationship between precision and recall under varying thresholds and is particularly suitable for scenarios with imbalanced class distributions. Similarly to the ROC curve, the area under the PR curve can also serve as a reference for model performance evaluation.

3. KS Curve and Optimal Threshold Selection:

While the ROC curve provides an overall assessment of model performance, the optimal classification threshold is determined using the KS curve. The Kolmogorov–Smirnov (KS) test, proposed by A.N. Kolmogorov and N.V. Smirnov, is based on cumulative distribution functions (CDFs) and is used to compare a sample with a reference probability distribution or to compare two samples.

4. DET Curve:

The Detection Error Tradeoff (DET) curve quantitatively characterizes the tradeoff between the False Negative Rate (FNR) and the False Positive Rate (FPR), providing a rigorous framework for evaluating binary classifiers. The DET curve plots FPR on the *x*-axis and FNR on the *y*-axis, where a lower FNR corresponds to a better detection performance. Unlike the ROC curve, the lower the DET curve is, the better is the predictive performance of the system.

5. Confusion Matrix:

The confusion matrix serves as a fundamental evaluation metric for classification models, providing a tabular representation of predicted versus actual class labels. This matrix structure comprises four critical components: true positives (TPs, correctly predicted positive instances), false positives (FPs, negative instances incorrectly classified as positive), false negatives (FNs, positive instances erroneously rejected), and true negatives (TNs, correctly identified negative cases). Particularly valuable for fault diagnosis in power equipment—where misclassification costs are substantial—the confusion matrix enables explicit quantification of model performance through specificity (TN/(FP + TN)) and sensitivity (TP/(TP + FN)) metrics, offering critical insights into a classifier’s discriminative capability across different fault types.

6. Accuracy and Precision:
  Accuracy measures the overall proportion of correct predictions made by the model, calculated as:
  (31) $$Accuracy=\frac{TP+TN}{TP+TN+FP+FN}$$
  Precision quantifies the proportion of true positive instances among all samples predicted as positive, computed as:
  (32) $$Precision=\frac{TP}{TP+FP}$$

### 4.4. Test Result Analysis

**Figure 3:**
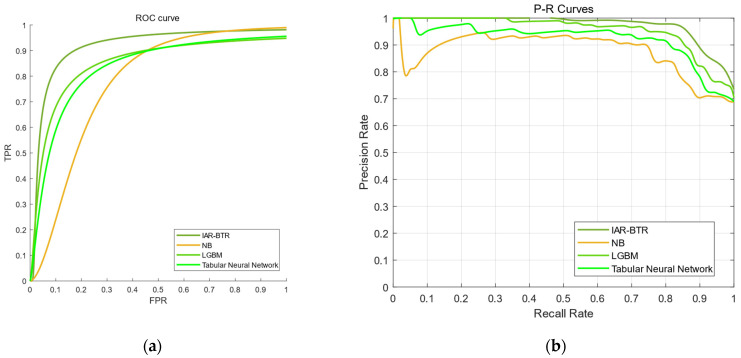
ROC and PR curves (**a**) ROC curves of IAR-BTR, LGBM, Tabular Neural Network and NB; (**b**) curves of IAR-BTR, LGBM, Tabular Neural Network and NB.

The ROC and PR curves were plotted for each method in Figure 3, and the AUC values of each model were calculated and listed in Table 4 and Table 5.

From the above simulation, it is evident that the IAR-BTR model proposed in this paper can achieve the highest fault diagnosis accuracy, with the AUC values of ROC and PR being 0.9137 and 0.8923, respectively. In contrast, the accuracy of other classifier models is lower, and the AUC values under the PR curve are roughly the same. It can also be inferred from the side that, when dealing with high-dimensional data and imbalanced data distribution, this method can achieve satisfactory results compared to existing ordinary machine learning methods.

**Figure 4:**
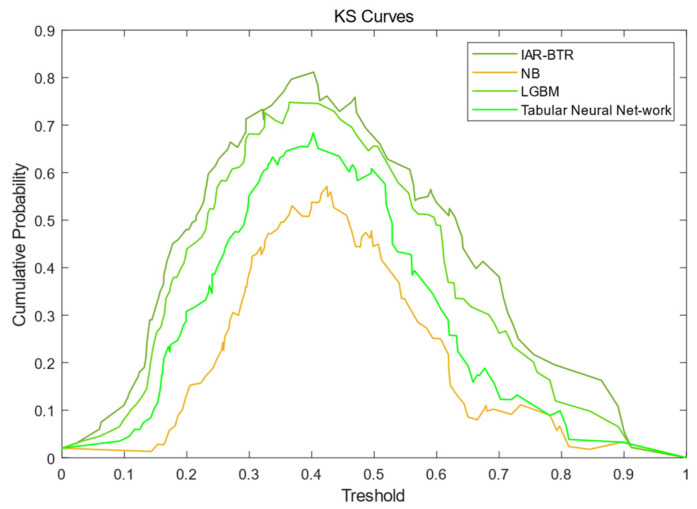
KS curves of IAR-BTR, LGBM, Tabular Neural Network and NB.

Figure 4 presents the KS curves of the IAR-BTR, LGBM, Tabular Neural Network and NB models in predicting the operational status of SF_6_ high-voltage circuit breakers on the test set, visually illustrating each model’s ability to distinguish between fault and non-fault states. KS values of each model were listed in Table 6. The KS curve of the IAR-BTR model exhibits the highest peak among the four, with a KS value of 0.8115—the highest among all methods—indicating that the incorporation of spatiotemporal non-stationary risk factors, through improved filtering thresholds and metric score calculations, significantly enhances the model’s discriminative capability.

**Figure 5:**
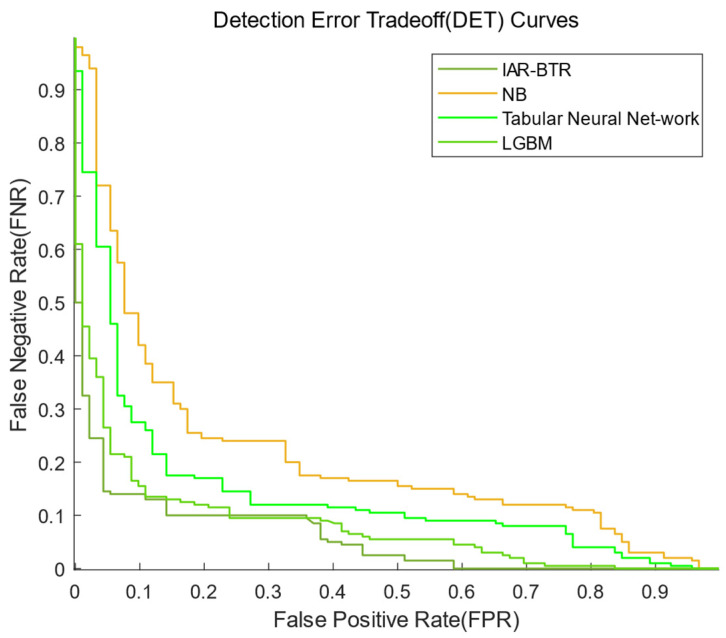
DET curves of the IAR-BTR, LGBM, Tabular Neural Network and NB models.

Figure 5 presents the DET curves of the IAR-BTR, LGBM, Tabular Neural Network and NB models in predicting the operational status of SF_6_ high-voltage circuit breakers on the test set. Analysis of the DET curve reveals that the IAR-BTR model’s curve is generally closest to the lower-left corner, indicating its superior ability to simultaneously reduce both the False Negative Rate (FNR) and the False Positive Rate (FPR). In this study, the IAR-BTR model achieves a significantly lower FNR and FPR compared to traditional approaches. This advantage stems from its environment-aware architecture, which effectively addresses the limitation of existing models that overlook external environmental factors. Future work will focus on quantifying the economic impact of reduced false alarms on power grid operation and maintenance.

**Figure 6:**
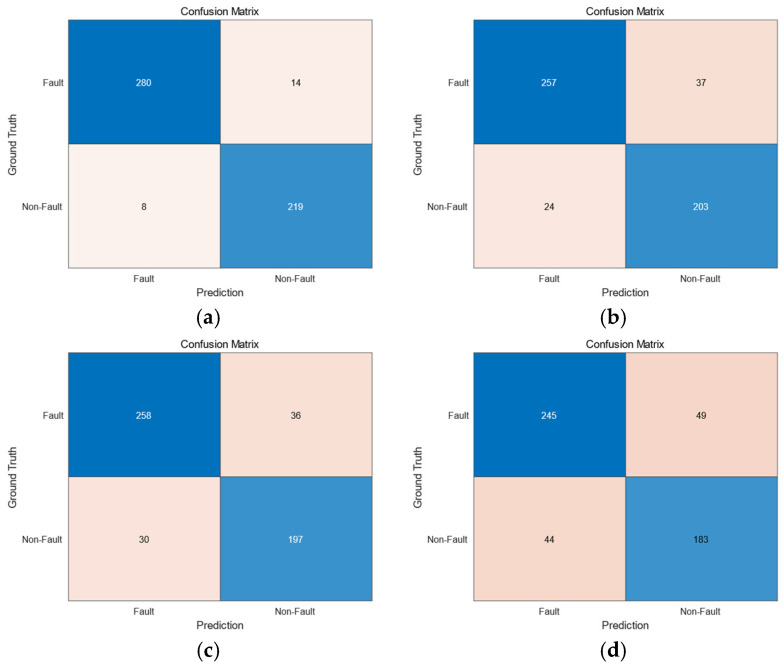
Confusion matrices of the four models (**a**) IAR-BTR (**b**) LGBM (**c**) Tabular Neural Network (**d**) NB.

Figure 6 Confusion matrices of the four models: (a) IAR-BTR, (b) LGBM, (c) Tabular Neural Network, (d) NB presents the confusion matrices of the four models, providing a visual representation of their classification performance. Based on the results, we quantitatively evaluated the model performance by calculating the accuracy and precision metrics for all four models, with detailed numerical results presented in Table 7.

Comparative analysis demonstrates the superior performance of our proposed method across all evaluation metrics. The proposed method achieved exceptional accuracy (95.78%) and precision (97.22%), correctly identifying 499 out of 521 data samples, with only 22 misclassified instances. The confusion matrix analysis further confirms the robustness and significant performance advantages of our proposed method over the three baseline models.

## 5. Limitations of the Model and Possible Future Directions

This study has certain limitations that should be acknowledged. First, due to experimental constraints, the proposed model was primarily compared with classical machine learning approaches, while more recent advanced algorithms were not included in the benchmarking analysis. Second, the model validation was conducted using circuit breaker operational data from a single substation, which may not fully represent the performance variations across different voltage levels (e.g., 750 kV and above) or diverse operating conditions. These limitations highlight the need for more comprehensive comparisons and broader validation in future research.

Future research directions will focus on three key enhancements to advance the state-of-the-art in circuit breaker condition monitoring: First, we will incorporate cutting-edge algorithms (e.g., transformer-based architectures and graph neural networks) to optimize multi-parameter fusion strategies through attention mechanisms and cross-modal feature learning. Second, in collaboration with national grid operators, we will establish a comprehensive validation framework using multi-voltage-level operational data (spanning 110 kV to 800 kV) to rigorously evaluate model generalizability across diverse infrastructure configurations. Third, we will develop an environmental adaptive module that dynamically integrates real-time correction factors for temperature, humidity, and other atmospheric variables, while further investigating the impacts of extreme natural disasters (including seismic events and flood conditions) on high-voltage circuit breaker degradation patterns through physics-informed machine learning approaches.

## 6. Conclusions

This paper proposes an Integrated Attribute-Weighted Risk Based on the Branch-Trunk Rule (IAR-BTR) model for evaluating the operational status of SF_6_ high-voltage circuit breakers, aiming to more accurately predict the spatiotemporal distribution of future faults. This facilitates timely maintenance interventions prior to failures, thereby minimizing their impact on power system operations. Specifically, the main contributions of this study are as follows:

1. Comprehensive consideration of internal and environmental factors affecting the operational status of SF_6_ high-voltage circuit breakers, enabling the model to effectively adapt to the increasing global frequency of extreme weather events and the growing environmental impact on equipment performance.
2. Improved association rule mining methodology to address the limitations of traditional methods in identifying spatiotemporally non-stationary factors during rare periods. By introducing filtering thresholds based on seasonal distributions and the importance of time segments, along with enhanced association metric calculations, the model effectively identifies critical low-probability seasonal factors strongly correlated with SF_6_ breaker failures.
3. Introduction of branch-trunk rule analysis to construct a multi-level, interpretable structural function model. By defining part failure weights and incorporating a normalization mechanism, the model enables system-wide risk perception and quantitative evaluation—from individual fault factors to overall system stability. This provides a theoretical foundation and algorithmic support for the monitoring and predictive maintenance of SF_6_ high-voltage circuit breakers.

The future research direction can further explore the impact of more external environmental factors (such as earthquakes, floods and other extreme natural disasters) on the operating state of high-voltage circuit breakers, and combine more intelligent algorithms (such as deep learning, reinforcement learning, etc.) to further improve the prediction performance of the model. In addition, with the further intelligence and digitalization of the power system, the progress of real-time data acquisition and processing technology will also provide more possibilities for the status evaluation of high-voltage circuit breakers.

## Figures and Tables

**Table 1:** Internal and external factors considered for the operational status assessment of SF_6_ high-voltage circuit breakers.

Internal Factors	External Environmental Factors
Mechanical performance	Temperature
Electrical performance	Humidity
Insulation performance	Icing intensity

**Table 2:** Runtime and memory consumption of the DFP-Growth algorithm and the FP-Growth algorithm.

Algorithm	Runtime	Memory Consumption
DFP-Growth	2135.44	156,553.34/311,455.12
FP-Growth	2784.94	156,553.34/311,455.12

**Table 3:** The key hyper parameter settings for IAR-BTR, LGBM, Tabular Neural Network and NB.

Model	Hyper Parameter	Value
IAR-BTR	$minspt\left(O\right)$	0.05
	$mincf\left(O\right)$	0.8
	$minlf\left(O\right)$	1.3
	$minJ\left(O\right)$	0.3
LGBM	num_leaves	31
	max_depth	−1
	min_data_in_leaf	20
	n_estimators	100
Tabular Neural Network	Learning Rate	1 × 10^−3^
	Hidden Layers	128
	Dropout Rate	0.4
	Batch Size	223
NB	priors	0.73
	var_smoothing	1 × 10^−9^

**Table 4:** AUC (ROC) values of IAR-BTR, LGBM, Tabular Neural Network and NB.

Model	AUC (ROC)%	Model	AUC (ROC)%
IAR-BTR	0.9137	Tabular Neural Network	0.8294
LGBM	0.8471	NB	0.7716

**Table 5:** AUC (PR) values of IAR-BTR, LGBM, Tabular Neural Network and NB.

Model	AUC (PR)%	Model	AUC (PR)%
IAR-BTR	0.8923	Tabular Neural Network	0.8086
LGBM	0.8274	NB	0.7843

**Table 6:** KS value of IAR-BTR, LGBM, Tabular Neural Network and NB.

Model	KS Value	Model	KS Value
IAR-BTR	0.8115	Tabular Neural Network	0.6837
LGBM	0.7480	NB	0.5711

**Table 7:** Accuracy and precision value of IAR-BTR, LGBM, Tabular Neural Network and NB.

Model	Accuracy	Precision
IAR-BTR	95.78	97.22
LGBM	88.29	91.46
Tabular Neural Network	87.33	89.58
NB	82.15	84.78

## Data Availability

The data presented in this study are available on request from the corresponding author. The data are not publicly available due to privacy or ethical restrictions.
